# The role of inflammatory cells in the association between helicobacter pylori and metabolic syndrome: a cross-sectional study

**DOI:** 10.3389/fpubh.2026.1913635

**Published:** 2026-10-07

**Authors:** Haiyan Yang, Liuying He, Ziqin Zeng, Nan Yang, Qian Wang, Haitao Guan, Ping Zhao

**Affiliations:** 1 Department of Gastroenterology, The Second Affiliated Hospital of Xi'an Jiaotong University, Xi'an, Shaanxi, China; 2 Department of Health Management, The Second Affiliated Hospital of Xi'an Jiaotong University, Xi'an, Shaanxi, China; 3 Department of Surgical Oncology, The Second Affiliated Hospital of Xian Jiaotong University, Xi'an, Shaanxi, China

**Keywords:** cross-sectional study, helicobacter pylori, inflammatory cells, mediation analysis, metabolic syndrome

## Abstract

**Background:**

*Helicobacter pylori* (*H. pylori*) infection may be related to metabolic disorders, with inflammation activation considered as a potential link. The study investigated the association between *H. pylori* infection and metabolic syndrome (MetS), and explored the potential role of inflammatory cells in this association.

**Methods:**

This study was conducted in a health check-up group from a tertiary hospital in northwest China. Logistic regression models were used to examine the association between *H. pylori* and MetS. Restricted cubic splines (RCS) were employed to examine both linear and nonlinear relationships between inflammatory markers and MetS. Additionally, the mediating role of inflammatory cells was examined.

**Results:**

The study comprised 20,491 participants (9,586 women, 10,905 men). 32.5% was the infection rate for *H. pylori*. Univariate analysis showed that MetS and its five components were significantly associated to *H. pylori* infection. After controlling for covariates, *H. pylori* infection was positively associated with MetS (OR: 1.27, 95% CI: 1.17–1.38). Furthermore, *H. pylori* infection was significantly associated with central obesity, hypertriglyceridemia, reduced high-density lipoprotein cholesterol, and elevated blood pressure (OR ranging from 1.11 to 1.17, all *p* < 0.05). However, no significant correlation was found between *H. pylori* infection and hyperglycemia. The RCS analysis showed that, with the exception of Monocyte-to-Lymphocyte Ratio (MLR), all other inflammatory markers show a nonlinear association with MetS. Exploratory mediation analysis suggested that leukocytes, lymphocytes, neutrophils, and monocytes accounted for 27.7%, 19.0%, 18.3%, and 6.9% of the statistical association between *H. pylori* infection and MetS, respectively.

**Conclusions:**

This study indicates that *H. pylori* infection was positively associated with MetS and its components (dyslipidemia, abdominal obesity, and elevated blood pressure). Inflammatory indices may be involved in this association.

## 1 Introduction

*Helicobacter pylori* (*H. pylori*) is a spiral-shaped Gram-negative bacterium that can colonize the epithelium of the gastric mucosa (1). *H. pylori* infection is a common chronic infection in humans worldwide. *H. pylori* is a widespread chronic infection affecting humans globally, but it is generally declining. Research indicates that developing countries have a higher infection rate compared to developed nations (2). A recent survey in China found the average infection rate of *H. pylori* to be 41.8%, with the highest infection rate of 63.6% in the northwest region of China (3). Several studies have found that *H. pylori* infection is associated with gastrointestinal diseases, such as chronic gastritis, peptic ulcers, and gastric cancer (4, 5). In addition, *H. pylori* has also been associated with various extra-gastrointestinal diseases, including osteoporosis, metabolic disorders, and non-alcoholic fatty liver disease (NAFLD) (6–8).

Metabolic Syndrome (MetS) is a common clinical syndrome that significantly raises the risk of cardiovascular diseases. It is characterized by abdominal obesity, high blood pressure, hyperglycemia, and dyslipidemia (9). Recently, the incidence of MetS has been rising due to factors such as an aging population, poor dietary habits, physical inactivity, and other unhealthy lifestyles (10). Although the definition of MetS slightly varies between countries and organizations, the differences are minimal. A study conducted in China from 2011 to 2015 reported a decreasing trend in MetS prevalence using most diagnostic criteria, except for the International Diabetes Federation (IDF) standard (11). In 2015, 32.97% of Chinese people aged 40 and above had MetS, with the northern regions showing the highest prevalence at 40.91% (12). Insulin resistance (IR) is the main underlying mechanism of MetS. It raises free fatty acid (FFA) levels, disrupts lipid metabolism, inhibits glucose uptake, and promotes oxidative stress and inflammation, all of which contribute to metabolic disturbances and accelerate the development of MetS (13).

Studies have shown that inflammation is commonly activated in individuals with MetS, often resulting in an elevated total white blood cell (WBC) count and their subtypes (14, 15). The increase in these immune cells not only serves as a marker of metabolic issues but may also be closely related to *H. pylori* infection. Research has found a positive link between *H. pylori* infection and the counts of neutrophil (NEU) and monocyte (MON) in peripheral blood (16). Both MetS and *H. pylori* infection correlate with alterations in inflammatory cells, suggesting that these cells may mediate the relationship between the two.

Given the inconsistent findings in existing studies and the lack of research in northwestern China, where *H. pylori* infection rates are high, this study aims to explore the association between *H. pylori* infection and MetS, as well as its components, in a population receiving health examinations at a tertiary hospital. Additionally, it examines the mediating role of inflammatory indices, providing a theoretical basis for the prevention and treatment of related diseases.

## 2 Methods

### 2.1 Population

We collected data from individuals who participated in the ^13^C-urea breath test (^13^C-UBT) at the Second Affiliated Hospital of Xi'an Jiaotong University between June 1, 2021, and May 31, 2023. In addition, we gathered demographic information, physical examination outcomes, and laboratory test data from this population, including a total of 21,817 individuals. The exclusion criteria are as follows: a history of partial or total gastrectomy (*n* = 3), a history of malignant tumors (*n* = 183), and incomplete data for one or more MetS components (*n* = 1140): missing waist circumference (WC) (*n* = 814), missing triglycerides (TG) (*n* = 178), missing high-density lipoprotein-cholesterol (HDL-C) (*n* = 15), missing systolic or diastolic blood pressure or history of hypertension (*n* = 101), and missing fasting plasma glucose (FPG) or history of diabetes mellitus (DM) (*n* = 32). After exclusions, 20,491 individuals remained for the final analysis (Figure 1). The Medical Ethics Committee of the Second Affiliated Hospital of Xi'an Jiaotong University approved this research (Ethics Approval Number: 2024177).

**Figure 1:**
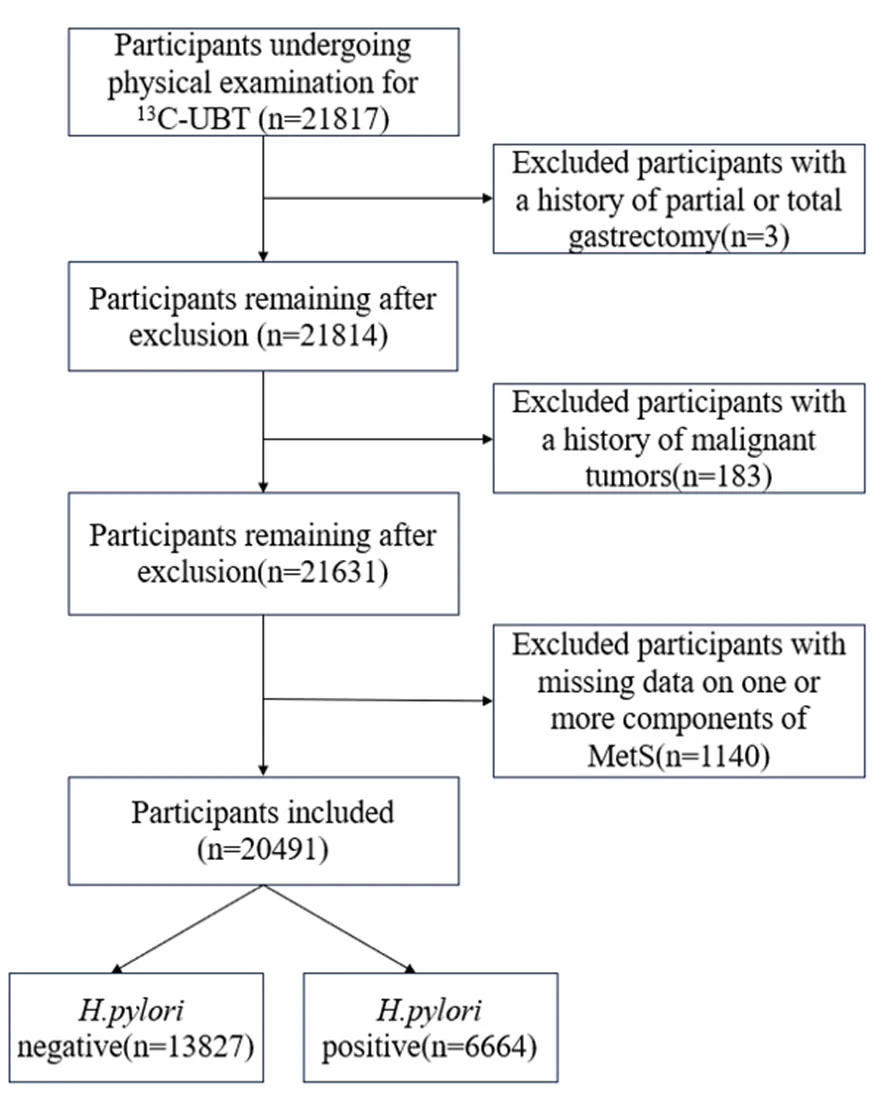
Flow chart for the research population. *n*, number; *H. pylori, Helicobacter pylori*.

### 2.2 Definition of *H. pylori* infection

In this study, the ^13^C-UBT was employed to detect *H. pylori* infection in participants, following a standardized sample collection procedure (17). All participants fasted for 3 h before exhaling to collect an initial breath sample. Next, they swallowed a ^13^C-urea capsule and stayed calm for 30 min, and exhaled again to collect secondary breath samples. The difference in the concentration ratio of ^13^CO2/^12^CO2 between the two gas samples was calculated, and the result was expressed as Delta Over Baseline (DOB). A DOB ≥ 4 indicates *H. pylori* positivity, while a DOB <4 indicates *H. pylori* negativity.

### 2.3 Definition of MetS

MetS is defined based on the Chinese Diabetes Society (CDS) criteria (18), which includes the following five components: central obesity (WC ≥90 cm for men, ≥85 cm for women); elevated blood pressure (≥130/85 mmHg or a self-reported history of hypertension); hyperglycemia (FPG ≥6.1 mmol/L or a self-reported history of DM); Hypertriglyceridemia(HTG)(fasting TG ≥1.7 mmol/L); and Reduced HDL-C (fasting HDL-C <1.04 mmol/L). Individuals meeting or exceeding three of these components are considered to have MetS.

### 2.4 Inflammation index

In this study, to investigate the role of hematological inflammatory indices in the association between *H. pylori* infection and MetS, we included counts of white blood cells (WBC), neutrophils (NEU), lymphocytes (LYM), and monocytes (MON), as well as inflammation-derived indices. These indices include the Monocyte-to-Lymphocyte Ratio (MLR) = monocyte count/lymphocyte count; the Neutrophil-to-Lymphocyte Ratio (NLR) = neutrophil count/lymphocyte count; and the Systemic Inflammation Response Index (SIRI) = neutrophil count × monocyte count/lymphocyte count.

### 2.5 Covariates

In this study, the following covariates were included for analysis. Include age, gender (male, female), marital status (single: unmarried, divorced, or widowed; married), education level (low: middle school or below, ≤ 9 years of education; middle: high school, technical school, or vocational school, 10–12 years of education; high: college or above, ≥13 years of education), occupation (physical labor, mental labor), smoking status (no: non-smokers or former smokers; yes: current smokers), and drinking status (no: non-drinkers or former drinkers; yes: current drinkers).

### 2.6 Statistical analysis

The R package and Empower Stats (version 4.2.0) were used to conduct the data analysis. Continuous variables were assessed for distribution normality. Normally distributed variables are presented as mean ± standard deviation, whereas skewed variables are presented as median and interquartile range (IQR). Categorical variables are expressed as frequencies and percentages (*n*, %). Multivariable logistic regression was used to examine the relationship between *H. pylori* infection and MetS, as well as its components. The regression analysis included three models with successive covariate adjustments: Model 1 was the univariate analysis; Model 2 adjusted for age and gender, while Model 3 additionally adjusted for smoking, drinking, education, occupation, and marital status. Subgroup analysis was performed to assess the robustness of the model. Additionally, restricted cubic spline (RCS) regression was applied to explore the nonlinear association between inflammatory markers and MetS. RCS models were fitted using four knots at the 5th, 35th, 65th, and 95th percentiles, with the median value of each inflammatory marker set as the reference. Finally, an exploratory mediation analysis was conducted to explore potential mediating factors between *H. pylori* infection and MetS. Each inflammatory marker was analyzed separately as a mediator due to the potential biological and statistical correlations among inflammatory cell counts. Mediation analyses were performed using the mediation R package (version 4.5.0). Results are presented as odds ratios (ORs) with 95% confidence intervals (CIs), and statistical significance was defined as a *p*-value less than 0.05.

## 3 Results

### 3.1 Description of the study population

This study involved 20,491 participants overall, with 46.8% women and 53.2% men. The infection rate for *H. pylori* was 32.5%, with men having a higher prevalence (56.41%) compared to women (43.59%). Based on *H. pylori* infection status, participants were classified into positive and negative groups (Table 1). Except for NLR, the two groups showed significant differences in all other variables (*P* < 0.05). Specifically, compared to the *H. pylori*-negative group, the *H. pylori*-positive group was older and had a higher proportion of individuals with obesity. Moreover, the *H. pylori*-positive group showed significantly higher levels of WC, blood pressure, FPG, blood lipids, and inflammatory cell counts. This group also had a greater proportion of smokers, drinkers, married individuals, those with lower educational attainment, physical laborers, and participants with MetS.

**Table 1:** Characteristics of the study population based on *H. pylori* infection.

Characteristics	*H. pylori* negative	*H. pylori* positive	*P*-value
	*n* = 13,827	*n* = 6,664	
Age (years)	37.82 ± 11.16	38.80 ± 11.18	<0.001^*^
Height (cm)	168.83 ± 8.34	169.21 ± 8.61	0.002^*^
Weight (Kg)	67.86 ± 13.43	69.59 ± 13.87	<0.001^*^
BMI (Kg/m^2^)	23.67 ± 3.55	24.18 ± 3.93	<0.001^*^
SBP (mmHg)	120.62 ± 14.22	122.12 ± 15.06	<0.001^*^
DBP (mmHg)	76.63 ± 10.32	77.77 ± 11.02	<0.001^*^
WC (cm)	80.89 ± 10.86	82.51 ± 11.23	<0.001^*^
FPG (mmol/L)	5.15 ± 0.91	5.21 ± 1.13	<0.001^*^
WBC(^*^10∧9/L)	5.65 (4.64–6.67)	5.90 (4.88–6.99)	<0.001^*^
NEU(^*^10∧9/L)	3.17 (2.41–3.86)	3.30 (2.55–4.10)	<0.001^*^
MON(^*^10∧9/L)	0.39 (0.31–0.46)	0.40 (0.33–0.48)	<0.001^*^
LYM(^*^10∧9/L)	1.89 (1.55–2.29)	1.96 (1.61–2.36)	<0.001^*^
NLR	1.66 (1.31–2.09)	1.68 (1.33–2.12)	0.488
MLR	0.20 (0.17–0.25)	0.20 (0.17–0.25)	0.009^*^
SIRI	0.64 (0.47–0.88)	0.66 (0.48–0.91)	0.019^*^
TG (mmol/L)	1.18 (0.82–1.76)	1.24 (0.84–1.85)	<0.001^*^
HDL-C (mmol/L)	1.28 (1.09–1.52)	1.28 (1.09–1.53)	<0.001^*^
Gender (*n*, %)			<0.001^*^
Men	7,146 (51.68%)	3,759 (56.41%)	
Women	6,681 (48.32%)	2,905 (43.59%)	
BMI (*n*, %)			<0.001^*^
<24	7,738 (55.96%)	3,314 (49.73%)	
24–28	4,517 (32.67%)	2,415 (36.24%)	
≥28	1,572 (11.37%)	935 (14.03%)	
Marital status (*n*, %)			<0.001^*^
Single	3,595 (26.00%)	1,464 (21.97%)	
Married	10,232 (74.00%)	5,200 (78.03%)	
Education level (*n*, %)			<0.001^*^
Low	2,072 (14.99%)	1,248 (18.73%)	
Middle	9,489 (68.63%)	4,436 (66.57%)	
High	2,266 (16.39%)	980 (14.71%)	
Occupation (*n*, %)			<0.001^*^
Physical labor	3,250 (23.50%)	1,726 (25.90%)	
Mental labor	10,577 (76.50%)	4,938 (74.10%)	
Smoking (*n*, %)			<0.001^*^
No	10,906 (78.87%)	4,992 (74.91%)	
Yes	2,921 (21.13%)	1,672 (25.09%)	
Drinking (*n*, %)			0.002^*^
No	7,531 (54.47%)	3,474 (52.13%)	
Yes	6,296 (45.53%)	3,190 (47.87%)	
DM (*n*, %)			0.022^*^
No	13,336 (96.45%)	6,384 (95.80%)	
Yes	491 (3.55%)	280 (4.20%)	
Hypertension (*n*, %)			<0.001^*^
No	11,800 (85.34%)	5,434 (81.54%)	
Yes	2,027 (14.66%)	1,230 (18.46%)	
Central obesity (*n*, %)			<0.001^*^
No	10,239 (74.05%)	4,597 (68.98%)	
Yes	3,588 (25.95%)	2,067 (31.02%)	
HTG (*n*, %)			<0.001^*^
No	10,133 (73.28%)	4,606 (69.12%)	
Yes	3,694 (26.72%)	2,058 (30.88%)	
Reduced HDL-C (*n*, %)			<0.001^*^
No	11,425 (82.63%)	5,258 (78.90%)	
Yes	2,402 (17.37%)	1,406 (21.10%)	
Elevated blood pressure (*n*, %)			<0.001^*^
No	9,335 (67.51%)	4,176 (62.67%)	
Yes	4,492 (32.49%)	2,488 (37.33%)	
Hyperglycemia (*n*, %)			0.017^*^
No	12,977 (93.85%)	6,196 (92.98%)	
Yes	850 (6.15%)	468 (7.02%)	
MetS (*n*, %)			<0.001^*^
No	11,700 (84.62%)	5,313 (79.73%)	
Yes	2,127 (15.38%)	1,351 (20.27%)	

Values are presented as mean ± SD, median (IQR), or *n* (%) as appropriate. ^*^P < 0.05; abbreviation: *H. pylori, Helicobacter pylori;* MetS, metabolic syndrome; DM, diabetes mellitus; BMI, body mass index. WC, waist circumference; TG, triglyceride; HTG, hypertriglyceridemia; HDL-C, high-density lipoproteins-cholesterol; WBC, white blood cell; NEU, neutrophils; MON, monocytes; LYM, Lymphocytes; SIRI, Systemic Inflammation Response Index; NLR, Neutrophil-to-Lymphocyte Ratio; MLR, Monocyte-to-Lymphocyte Ratio.

### 3.2 Relationship between *H. pylori* infection and MetS.

Table 2 presents the association between *H. pylori* infection and MetS. After adjustment for potential confounders, *H. pylori* infection was significantly associated with MetS. (OR 1.27, 95% CI 1.17, 1.38).

**Table 2:** The association between *H. pylori* infection and MetS and its components among the participants.

Outcome	Model 1		Model 2		Model 3	
	OR (95% CI)	*P*-value	OR (95% CI)	*P*-value	OR (95% CI)	*P*-value
MetS						
No	1.0		1.0		1.0	
Yes	1.40 (1.30, 1.51)	<0.0001^*^	1.30 (1.20, 1.41)	<0.0001^*^	1.27 (1.17, 1.38)	<0.0001^*^
Central obesity						
No	1.0		1.0		1.0	
Yes	1.28 (1.20, 1.37)	<0.0001^*^	1.19 (1.11, 1.28)	<0.0001^*^	1.17 (1.09, 1.25)	<0.0001^*^
HTG						
No	1.0		1.0		1.0	
Yes	1.23 (1.15, 1.31)	<0.0001^*^	1.14 (1.06, 1.22)	0.0002^*^	1.11 (1.04, 1.19)	0.0020^*^
Reduced HDL-C						
No	1.0		1.0		1.0	
Yes	1.27 (1.18, 1.37)	<0.0001^*^	1.20 (1.11, 1.29)	<0.0001^*^	1.17 (1.08, 1.26)	<0.0001^*^
Elevated blood pressure						
No	1.0		1.0		1.0	
Yes	1.24 (1.16, 1.32)	<0.0001^*^	1.15 (1.07, 1.23)	<0.0001^*^	1.15 (1.08, 1.23)	<0.0001^*^
Hyperglycemia						
No	1.0		1.0		1.0	
Yes	1.15 (1.03, 1.30)	0.0168^*^	1.04 (0.92, 1.17)	0.5698	1.03 (0.91, 1.16)	0.6428

Model 1, univariate analysis; Model 2, adjusted for age and gender; Model 3, further adjusted for smoking, drinking, education level, occupation, and marital status. ^*^P < 0.05; OR, odds ratio; CI, confidence interval; *H. pylori, Helicobacter pylori;* MetS, metabolic syndrome; HTG, hypertriglyceridemia; HDL-C, high-density lipoproteins-cholesterol.

Additionally, we examined the association between *H. pylori* infection and each component of MetS (Table 2). After adjusting for covariates, *H. pylori* infection was positively associated with central obesity (OR 1.17, 95% CI 1.09–1.25), hypertriglyceridemia (OR 1.11, 95% CI 1.04–1.19), reduced HDL-C (OR 1.17, 95% CI 1.08–1.26), and elevated blood pressure (OR 1.15, 95% CI 1.08–1.23). However, no significant association was observed between *H. pylori* infection and hyperglycemia after full adjustment (OR 1.03, 95% CI 0.91–1.16, *P* = 0.6428).

### 3.3 Subgroup analysis

To determine whether the association between *H. pylori* infection and MetS differed across subgroups, we carried out a subgroup analysis (Figure 2). The association between *H. pylori* infection and MetS was observed among individuals aged <60 years, male, female, those with lower/middle education levels, physical laborers, and mental laborers (OR 1.28, 95% CI 1.18–1.39; OR 1.23, 95% CI 1.13–1.35; OR 1.48, 95% CI 1.22–1.78; OR 1.25, 95% CI 1.05–1.48; OR 1.30, 95% CI 1.18–1.44; OR 1.28, 95% CI 1.09–1.50; OR 1.26, 95% CI 1.15–1.39, respectively). However, none of the subgroup variables showed significant interaction effects (all *P*-interaction >0.05).

**Figure 2:**
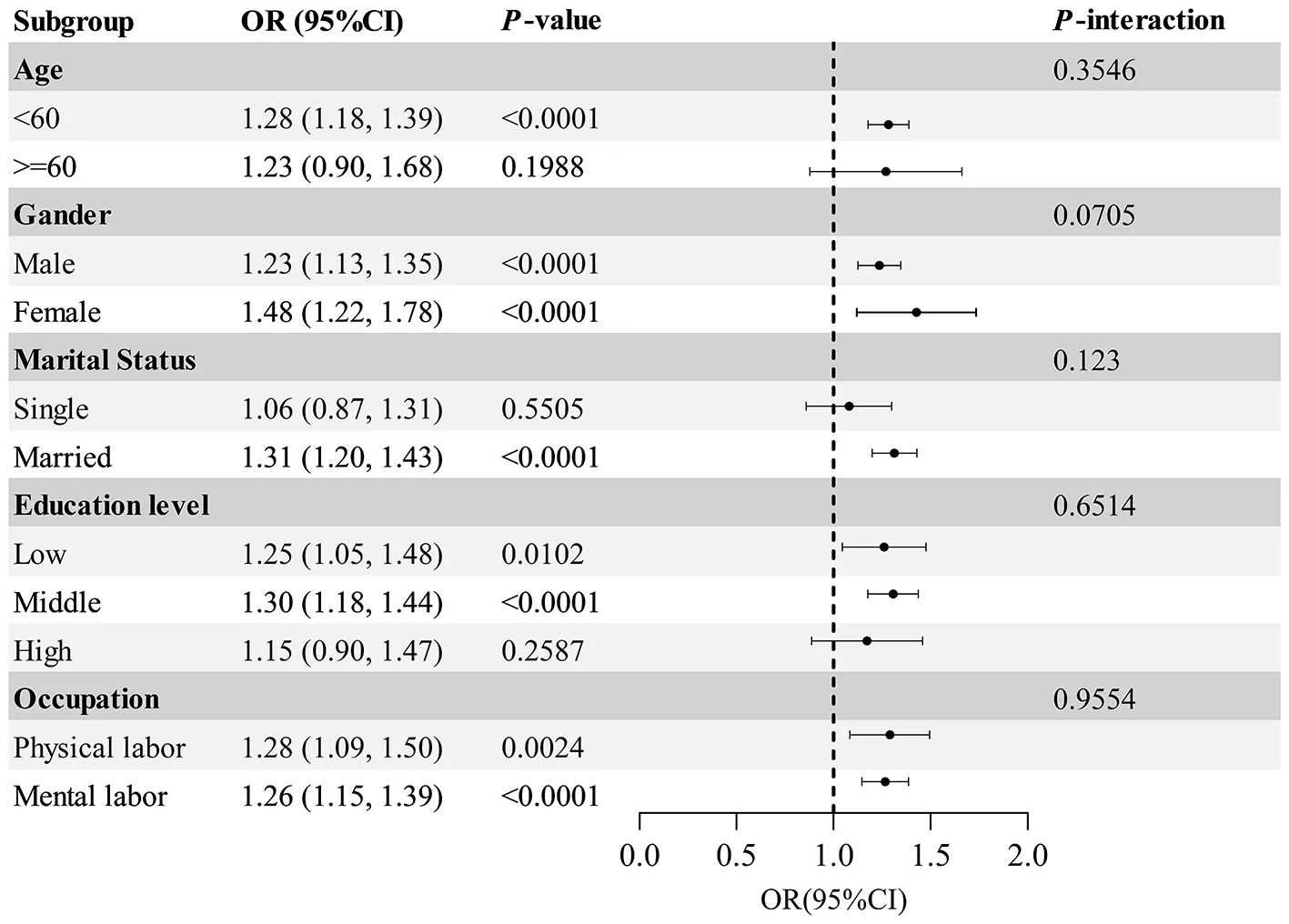
Subgroup analysis of the association between *H. pylori* infection and MetS. Adjusted for gender, age, smoking, drinking, education level, occupation, and marital status. OR, odds ratio; CI, confidence interval.

### 3.4 RCS analysis of the association between inflammatory markers and MetS

This study used RCS models to evaluate the dose–response relationships between inflammatory indicators and MetS. After adjustment for age, sex, marital status, education level, smoking status, and alcohol consumption, significant associations were observed between WBC and their subtypes (NEU, MON and LYM) and MetS (Figure 3). These associations showed significant nonlinear monotonic increasing trends (all *P* for overall <0.001, *P* for nonlinear <0.001).

**Figure 3:**
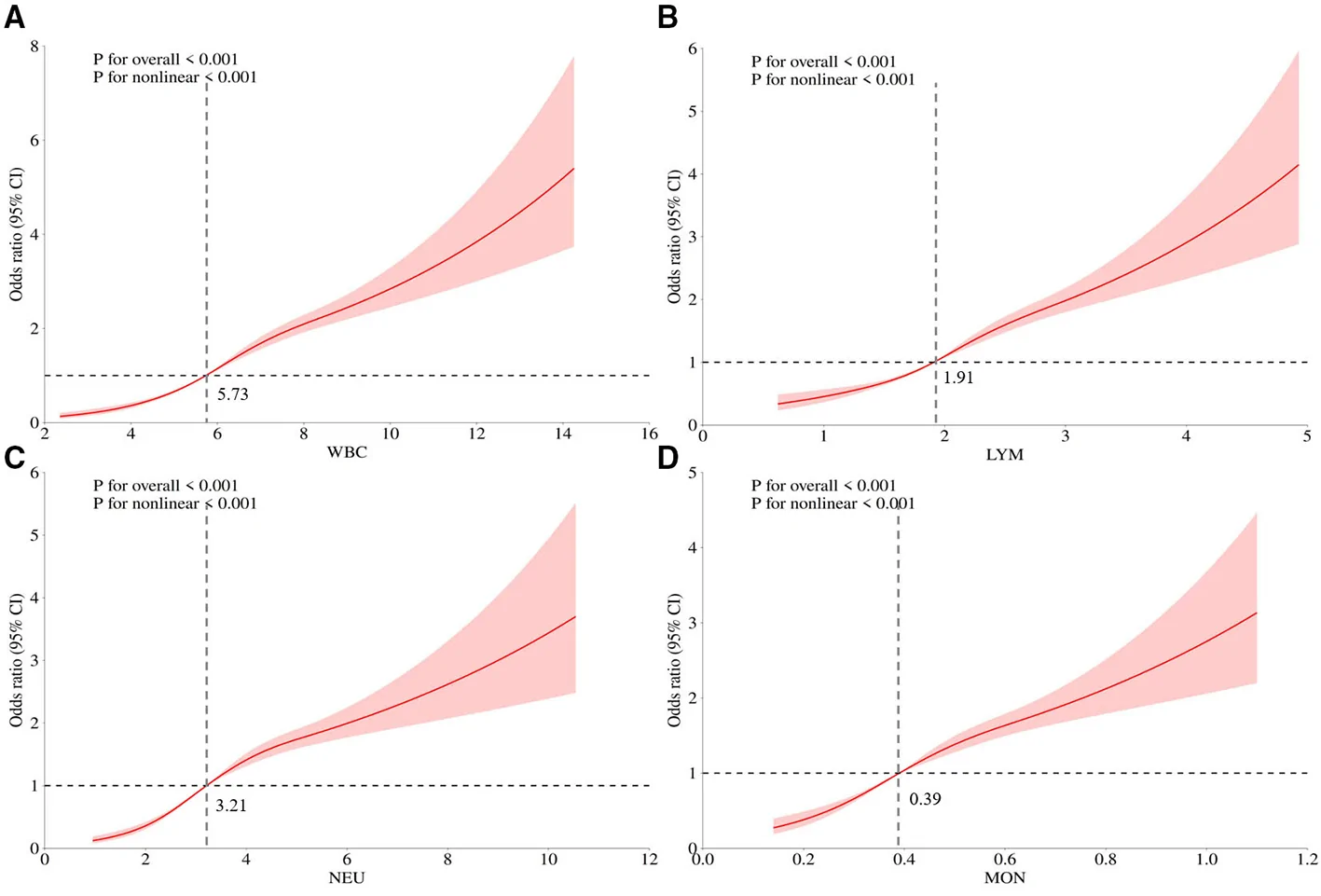
RCS analysis of the association between Inflammatory cells and MetS. **(A)** RCS analysis of the association between WBC and MetS; **(B)** RCS analysis of the association between LYM and MetS; **(C)** RCS analysis of the association between NEU and MetS; **(D)** RCS analysis of the association between MON and MetS. Adjusted for gender, age, smoking, drinking, education level, occupation, and marital status. RCS, restricted cubic spline; WBC, white blood cell; NEU, neutrophils; MON, monocytes; LYM, lymphocytes.

In addition, among the composite inflammatory indicators (Figure 4), both NLR and SIRI showed significant nonlinear associations with MetS. However, no significant association was observed between MLR and MetS, and the nonlinear relationship was also not significant (*P* for overall = 0.154, *P* for nonlinear = 0.501).

**Figure 4:**
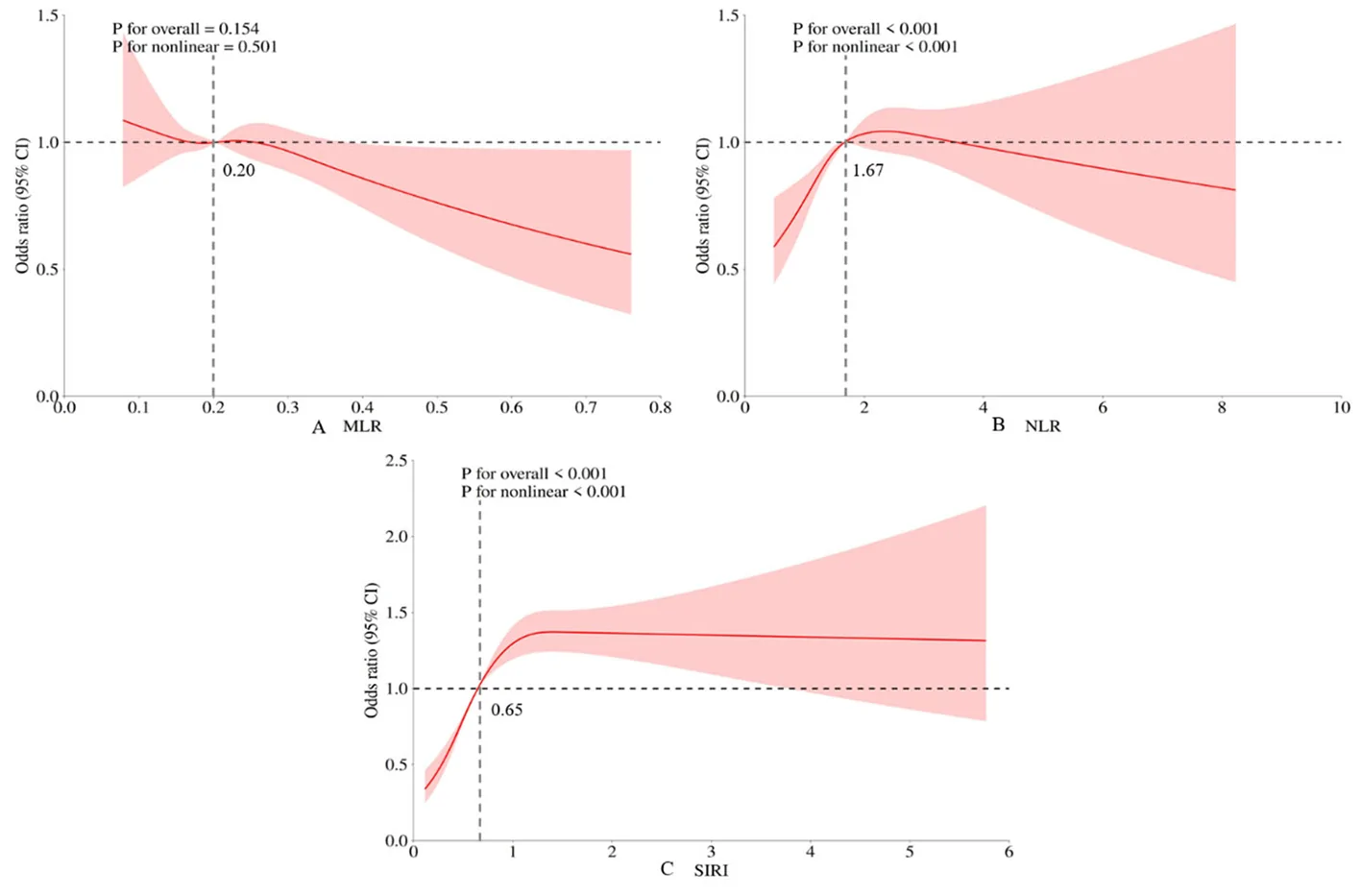
RCS analysis of the association between composite inflammatory markers and MetS. **(A)** RCS analysis of the association between MLR and MetS; **(B)** RCS analysis of the association between NLR and MetS; **(C)** RCS analysis of the association between SIRI and MetS. Adjusted for gender, age, smoking, drinking, education level, occupation, and marital status. RCS, restricted cubic spline; SIRI, systemic Inflammation Response Index; NLR, neutrophil-to-lymphocyte ratio; MLR, monocyte-to-lymphocyte ratio.

### 3.5 Mediation analysis through inflammatory indices

This study explored the possible mediating role of hematological inflammatory indices in the link between *H. pylori* infection and MetS (Table 3). In this analysis, *H. pylori* infection was the independent variable, inflammatory indices served as mediators, and MetS was the dependent variable. The results indicated that WBC, NEU, MON, and LYM partially mediated the association between *H. pylori* infection and MetS (all *p* < 0.001), with WBC showing the strongest mediating effect, accounting for 27.7%. This was followed by LYM and NEU, accounting for 19.0% and 18.3%, respectively, while MON had the smallest mediating effect at 6.9% (Figure 5). The total effects of the composite inflammatory indices, including the NLR, MLR, and SIRI, were significant, but their mediating effects were not.

**Table 3:** Exploratory mediation analysis of inflammatory indices in the association between *H. pylori* infection and MetS.

Characteristic	Total effect	Mediation effect	Direct effect	Proportion mediated
	β (95% CI)	β (95% CI)	β (95% CI)	β (95% CI)
WBC	0.030 (0.019, 0.041) <0.0001	0.008 (0.006, 0.010) <0.0001	0.022 (0.011, 0.032) <0.0001	0.277 (0.196, 0.425) <0.0001^*^
NEU	0.031 (0.020, 0.041) <0.0001	0.006 (0.004, 0.007) <0.0001	0.025 (0.015, 0.036) <0.0001	0.183 (0.125, 0.285) <0.0001^*^
MON	0.030 (0.019, 0.041) <0.0001	0.002 (0.001, 0.003) <0.0001	0.028 (0.018, 0.039) <0.0001	0.069 (0.028, 0.125) <0.0001^*^
LYM	0.029 (0.018, 0.039) <0.0001	0.006 (0.004, 0.007) <0.0001	0.024 (0.013, 0.034) <0.0001	0.190 (0.130, 0.302) <0.0001^*^
NLR	0.030 (0.020, 0.041) <0.0001	0.000 (−0.000, 0.000) 0.9020	0.030 (0.020, 0.041) <0.0001	0.000 (−0.008, 0.008) 0.9020
MLR	0.030 (0.019, 0.041) <0.0001	0.000 (−0.000, 0.001) 0.0680	0.030 (0.019, 0.040) <0.0001	0.008 (−0.001, 0.023) 0.0680
SIRI	0.031 (0.020, 0.041) <0.0001	0.000 (−0.000, 0.001) 0.2200	0.030 (0.019, 0.041) <0.0001	0.011 (−0.009, 0.033) 0.2200

Adjusted for gender, age, smoking, drinking, education level, occupation, and marital status. ^*^P < 0.05; β, Estimate; WBC, white blood cell; NEU, neutrophils; MON, monocytes; LYM, Lymphocytes; NLR, Neutrophil-to-Lymphocyte Ratio; SIRI, Systemic Inflammation Response Index; MLR, Monocyte-to-Lymphocyte Ratio.

**Figure 5:**
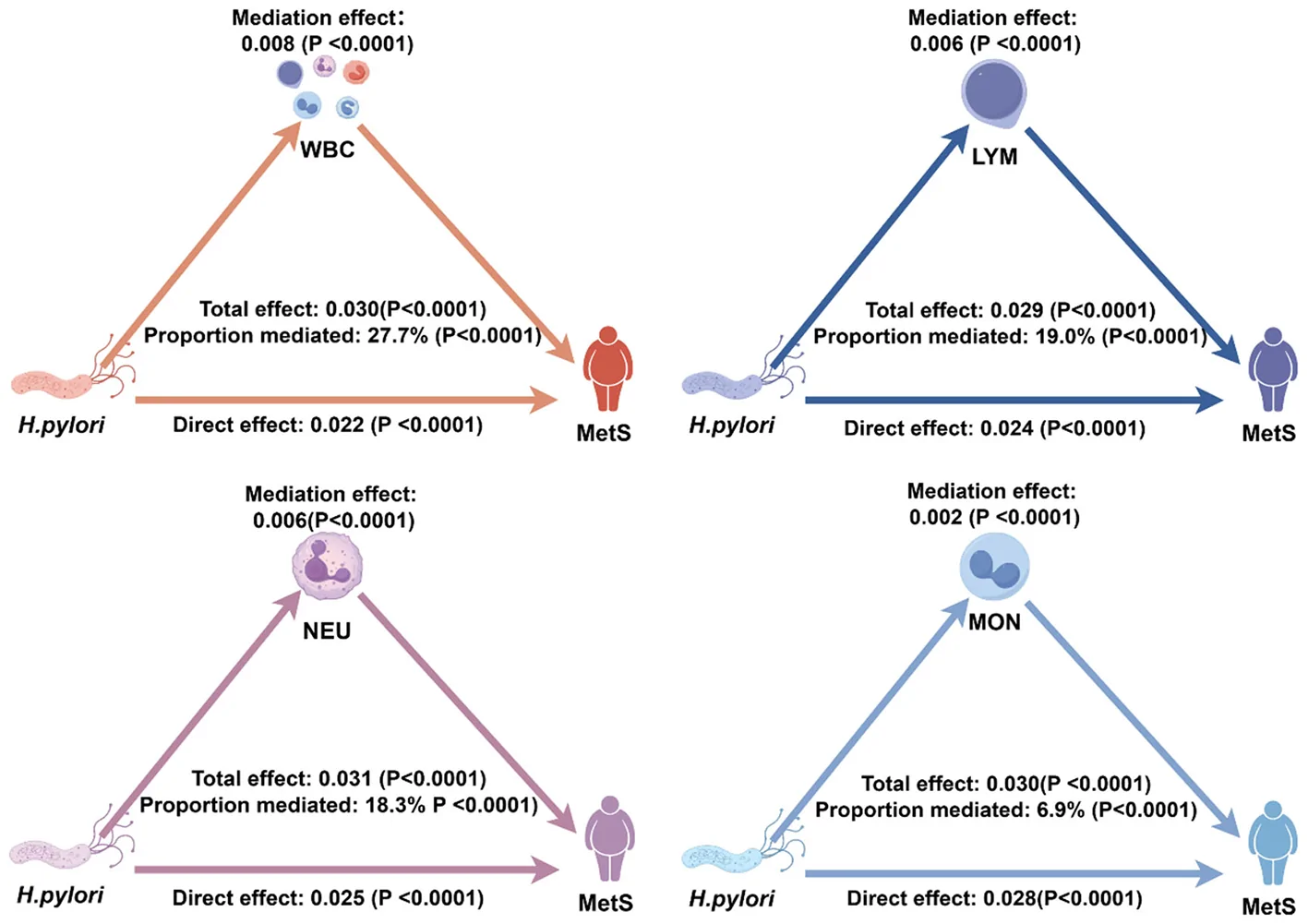
Exploratory mediation analysis of inflammatory cell counts in the association between H. pylori infection and MetS. The figure elements were sourced by Figdraw. *H. pylori, Helicobacter pylori;* MetS, metabolic syndrome; WBC, white blood cell; NEU, neutrophils; MON, monocytes; LYM, Lymphocytes.

## 4 Discussion

This study investigated the association between *H. pylori* infection and MetS using data from a health check-up population. After adjusting for covariates, logistic regression analysis revealed a significant positive association between *H.pylori* infection and MetS. Stratified analysis further demonstrated that the association between *H. pylori* infection and MetS was observed among individuals aged <60 years, males, those with lower education levels, and physical laborers.

Among the MetS components, central obesity, HTG, reduced HDL-C, and elevated blood pressure were positively correlated with *H. pylori* infection, while no significant association was found with hyperglycemia.

The meta-analysis conducted by Sadeghi et al. found the *H. pylori* infection rate in obese populations to be 32.3% (19). A cross-sectional study by Chen et al. revealed that central obesity, as defined by the waist-to-height ratio (WHtR), was significantly linked to *H. pylori* infection in individuals aged ≤ 50, while obesity based on BMI was not (20). Studies suggest that central obesity is crucial in the development of IR by influencing the secretion of several cytokines like IL-1, IL-6, IL-17, and TNF-α (21). Adiponectin has insulin-sensitizing effects, and its blood concentration is negatively correlated with visceral fat content (22), while Leptin is pivotal in controlling energy intake and expenditure. Individuals with *H. pylori* infection generally have lower levels of leptin, and alterations in this adipokine may increase food intake or uneven fat distribution, contributing to obesity (23).

Chen et al. revealed a notable connection between *H. pylori* infection and HTG (24). Research conducted by Li et al. revealed that DM individuals with *H. pylori* had lower HDL-C levels compared to those without the infection, while TG levels were similar in both groups (25). Multiple studies have confirmed that *H. pylori* infection can lead to lipid abnormalities, which is consistent with our findings. The main mechanisms include: on one hand, *H. pylori* infection activates inflammatory factors, such as IL-6, TNF-α, and CRP, intensifying oxidative stress and inducing IR, which disrupts hepatic lipid metabolism and causes uneven fat distribution; on the other hand, *H. pylori* infection may also reduce insulin secretion by affecting pancreatic β-cells (26). IR is acknowledged as a primary mechanism in metabolic dysfunction, leading to reduced sensitivity to insulin, weakening its inhibitory effect on adipose tissue, and impairing the liver's ability to transport and absorb various lipid components, ultimately causing abnormal serum lipid profiles (27).

We discovered a positive link between high blood pressure and *H. pylori* infection, similar to what Yang et al reported (28). The main mechanisms underlying hypertension include endothelial dysfunction and improper activation of the renin-angiotensin-aldosterone system (RAAS) (29). *H. pylori* infection can activate various pro-inflammatory cytokines, inducing a chronic low-grade inflammatory state. This inflammation stimulates endothelial cells, disrupting normal vascular contraction and relaxation, and impairing the self-regulation of blood pressure (30). Furthermore, *H. pylori* infection induces IR, leading to lipid metabolism disorders characterized by HTG, high LDL-C, and low HDL-C. These changes promote lipid deposition in the vascular endothelium, triggering or exacerbating atherosclerosis, increasing vascular stiffness, and ultimately raising blood pressure (31). *H. pylori* infection also damages the gastrointestinal mucosa, affecting absorption and digestion. *H. pylori* infection has been found to impair the absorption of folate, vitamin B12, and other substances, leading to elevated homocysteine (Hcy) levels. Hcy can promote cardiovascular damage through several mechanisms (32). These pathological changes contribute to the development and progression of atherosclerosis, ultimately increasing blood pressure.

You et al. found that, in non-diabetics, those with persistent *H. pylori* infection had significantly higher blood glucose and glycosylated hemoglobin levels compared to the *H. pylori*-negative group (33). However, no significant association between hyperglycemia and *H. pylori* infection was observed in our study. This finding may be partly explained by the relatively healthier status of the health check-up population, where individuals with metabolic abnormalities may have received earlier diagnosis and management. The inflammatory response and oxidative stress caused by *H. pylori* infection are key drivers of IR, which disrupts glucose homeostasis and leads to blood glucose abnormalities (34). Additionally, studies have shown that *H. pylori* colonization can disrupt the gut microbiota, potentially altering dietary patterns, exacerbating glucose metabolism imbalance (35).

Additionally, inflammatory cells (WBC and their subtypes) may partially explain the positive association between *H. pylori* infection and MetS.

Jiao et al. reported that WBC counts were significantly elevated in individuals infected with *H. pylori* (36). This is further supported by the findings of Kondo et al., who demonstrated a substantial decrease in the total counts of peripheral WBCs, neutrophils, and monocytes following *H. pylori* eradication (16). These findings suggest that *H. pylori* infection may serve as an initiating factor for the elevation of circulating inflammatory cell levels. Mechanistically, lipopolysaccharide (LPS) and virulence factors (such as CagA) released by *H. pylori* strains act as pathogen-associated molecular patterns (PAMPs) to activate host pattern recognition receptors (PRRs). This activation triggers specific signaling pathways, inducing the expression and secretion of pro-inflammatory cytokines (such as TNF-α and IL-6) and various chemokines (37). Beyond local recruitment of immune cells, these inflammatory mediators enter the systemic circulation and stimulate bone marrow hematopoiesis. This results in the sustained release of neutrophils and monocytes into the peripheral blood, thereby maintaining a state of chronic low-grade inflammation (38).

Previous studies have established that chronic low-grade inflammation serves as a critical bridge between bacterial infection and metabolic dysfunction, driving metabolic disorders such as IR and dyslipidemia through multiple mechanisms (39). During this process, neutrophils exacerbate oxidative stress and impair vascular endothelial function via the release of elastase and reactive oxygen species (ROS) (40); while lymphocytes mediate complex adaptive immune responses; collectively, these cellular activities form the pathological basis for hypertension and lipid metabolic abnormalities (41). Concurrently, circulating monocytes infiltrate adipose tissue and differentiate into pro-inflammatory macrophages. These macrophages secrete inflammatory mediators that disrupt insulin receptor substrate (IRS) phosphorylation, thereby directly inducing insulin resistance and ultimately leading to impaired glucose metabolism and obesity (42).

Overall, the activation and systemic circulation of inflammatory cells may represent a potential linking between *H. pylori* infection and MetS.

This study provides preliminary clinical insights into the association between *H. pylori* infection and MetS by demonstrating a positive association and conducting an exploratory mediation analysis to assess the potential role of inflammatory cells. However, several limitations exist. First, as a cross-sectional study based on a Chinese health check-up population, this study cannot establish causality or temporal association between *H. pylori* and MetS. Therefore, the mediation analysis in this paper is an exploratory analysis rather than evidence of a causal pathway. Second, the ^13^C-UBT mainly reflects current active *H. pylori* infection but cannot distinguish previous exposure, chronic infection, or eradication history. Due to the lack of serological markers (such as anti-*H. pylori* antibodies) and information on previous *H. pylori* eradication therapy, we were unable to further evaluate the potential effects of different infection statuses and treatment history. Third, although inflammatory indices, including NLR, MLR, and SIRI, were used as surrogate markers of systemic inflammation, direct inflammatory biomarkers such as CRP, IL-6, IL-1β, and TNF-α were not available in our database. Fourth, participants with missing data were excluded rather than imputed, which may have introduced potential selection bias, although the proportion of missing data was relatively small. Finally, residual confounding may exist due to unavailable lifestyle and clinical information, including diet, physical activity, socioeconomic status, and medication use. In addition, some statistically significant baseline differences may have limited clinical relevance due to the large sample size. Further prospective studies with comprehensive clinical and inflammatory assessments are needed to validate our findings.

## 5 Conclusion

This cross-sectional study showed that *H.pylori* infection is positively associated with MetS and its components (central obesity, hypertriglyceridemia, low HDL-C levels, and elevated blood pressure). Additionally, exploratory mediation analysis indicated that inflammatory cells, particularly WBC and their subtypes, partially explain the positive association between *H. pylori* and MetS.

## Data Availability

The original contributions presented in the study are included in the article/supplementary material, further inquiries can be directed to the corresponding authors.
